# Engorgement of the Uterus

**Published:** 1843-03

**Authors:** 


### Selections from American and Foreign Journals.

---

*Engorgement of the Uterus.*—In a pamphlet published by Doctor Clement Ollivier, of Angers, on the treatment of prolapsus uteri, he speaks strongly against the use of differently shaped pessaries, which are employed indiscriminately, without paying attention to the cause of the prolapsus, which, according to Dr. Ollivier, is nothing more than an engorgement. Thence arise the symptoms which are constantly observed, and which are attributed to any cause other than the presence of a foreign body, and its contact with a painful and inflamed surface.

Ollivier considers that one of the most frequent causes of this affection in young girls, with whom it is very rare, is masturbation. He says, that one of the most frequent causes of chronic engorgement of the uterus in virgins, or women who do not have any communication with men, is masturbation, which, by gradually inducing disorder in the uterine functions, gives rise at first to spasm of the organ, which affects the secretion of the menstrua; on the other hand, this excitement, if frequently repeated, finally brings on a more or less intense sanguineous congestion, which gives rise to a kind of impermeability of the uterine parenchyma, caused by a slight inflammatory affection; then the dysmenorrhœa, at a later period, becoming habitual, induces amenorrhœa, which ultimately determines more dangerous diseases. Sterility is always an inevitable result, unless the diseased state of the uterus being arrested, allows those portions of the viscus which continue healthy to perform their functions; the catamenia may then reappear, but are almost always accompanied by uterine colics; the matrix may recover its powers of conception, but during gestation a period arrives when the uterus, not being able to enlarge freely, on account of the inflammatory action it has undergone before conception, reacts upon the product it contains, and almost always determines an abortion; in this way the pregnancies of women affected with morbid conditions of the uterus almost always terminate.

Masturbation, in causing a disordered condition of the entire uterus, produces more frequently an engorgement of the body of the organ rather than of the neck, whilst an exactly contrary condition obtains in women who have connection with men. In virgins the affection of the body of the uterus is more frequently found, that of the cervix uteri more rarely.

Ollivier mentions, among other causes of engorgement of the uterus, the irritation of the sexual organs by primary connection, a cause of irritation of the organ the more dangerous, that it has hitherto escaped the notice of medical men, either because they do not attach sufficient importance to it, or because women conceal from them the knowledge of their illness, notwithstanding the sufferings they endure.

The dysmemorrhœa, which almost always follows abortions, is the result of an inflammatory engorgement more or less considerable, and susceptible of cure; this engorgement is the cause of the sterility that follows miscarriages. The frequency of these inflammatory engorgements observed by the vulgar has rendered abortions more dangerous in their eyes than a delivery at the full period; when they take place during the first pregnancy, they are the more frequently to be attributed to a too great sensibility of the uterus, as yet unaccustomed to the sensations produced by coition. It is this sensibility which gives rise to consecutive inflammatory symptoms; under other circumstances this uterine sensibility causes the disorders which precede menstruation.

Ollivier attributes the sterility which occurs to most women in large towns, after their first and second labors, to a similar cause. The editors of the "Journal de Medicine et de Chirurgie Pratiques" observe, with respect to this opinion, that they agree with Ollivier, that the engorgement of the uterus may sometimes prevent conception, but that another cause for this pretended sterility in great towns, and Paris especially, must be sought for. Considerations of a different kind will explain the small number of children found in families, whose pecuniary means are not in just relation with their daily expenses.—*Med. Examiner*, from *Provincial Medical Journal.*

